# Pulmonary Hypertension in Women

**DOI:** 10.14797/mdcvj.1308

**Published:** 2024-03-14

**Authors:** Eunwoo Park, Zeenat Safdar

**Affiliations:** 1 Houston Methodist Hospital, Houston, Texas, US; 2 Houston Methodist Lung Center, Houston Methodist Hospital, Houston, Texas, US; 3 Weill Cornell College of Medicine, New York, New York, US

**Keywords:** pulmonary hypertension, pulmonary arterial hypertension, heart disease in women, estrogen paradox

## Abstract

Pulmonary arterial hypertension (PAH) is a rare devastating disease characterized by elevated pulmonary artery pressure and increased pulmonary vascular resistance. Females have a higher incidence of PAH, which is reflected globally across registries in the United States, Europe, and Asia. However, despite female predominance, women had better outcomes compared with male patients, a finding that has been labeled the “estrogen paradox.” Special considerations should be given to women with PAH regarding sexual health, contraception, family planning, and treatment before, during, and after pregnancy. Pregnant women with PAH should be referred to a pulmonary hypertension care center; a multidisciplinary team approach is recommended, and Cesarean section is the preferred mode of delivery. While pregnancy outcomes have improved over the years with PAH-specific therapy, pregnancy portends a high-risk for those with PAH. Continued research is needed to tailor PAH treatment for women.

## Introduction

**Figure 1:**
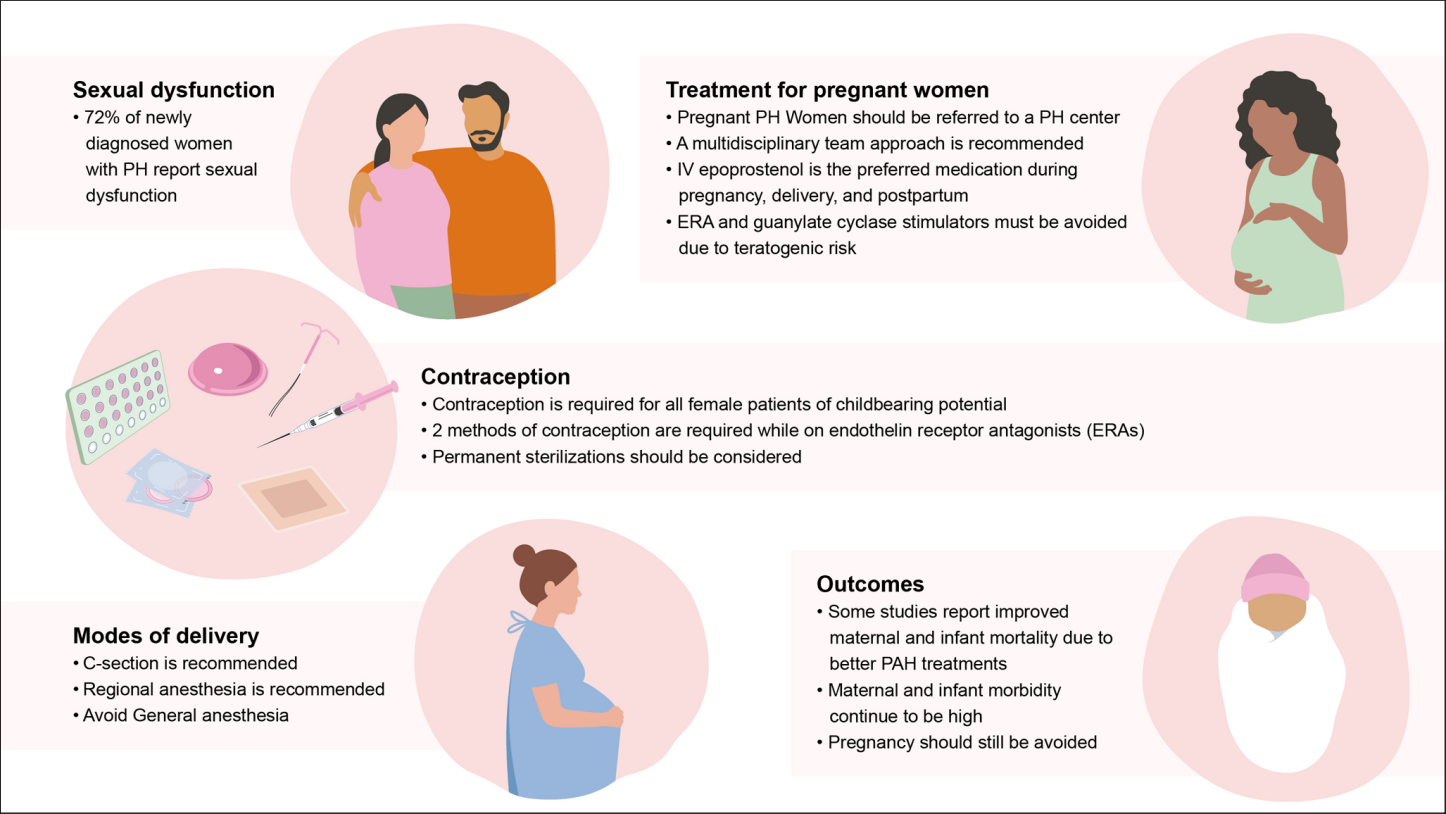
Overview of the unique challenges faced by women with pulmonary hypertension. PH: pulmonary hypertension; PAH: pulmonary arterial hypertension; IV: intravenous

Pulmonary hypertension (PH) is a rare devastating disease characterized by elevated pulmonary artery pressure and increased pulmonary vascular resistance.^1^ Pulmonary hypertension was first classified in 1973 at the 1st World Symposium on Pulmonary Hypertension, and since then the classification system has been revised multiple times.^2^ Recently, the definition of PH was expanded to include a mean pulmonary artery pressure (mPAP) > 20 mm Hg on supine right heart catheterization (RHC) to aid in early detection of disease.^1,3^ PH is divided into five different groups, where Group 1 (those with pulmonary arterial hypertension, or PAH) is known to predominantly affect women.^3,4^ Symptoms include fatigue, bendopnea, and dyspnea, especially with minor exertion.^1,3^ Women with PH are faced with unique challenges, and this article focuses on the pathophysiology, treatment, and outcomes in female PH patients, with a special focus on PAH due to its female predominance (Figure 1).

## Epidemiology

PH affects all age groups, with an estimated prevalence of 1% of the world’s population.^3^ The prevalence is higher in patients aged > 65 years due to increased cardiac and pulmonary etiologies, with lung disease and chronic obstructive pulmonary disease (COPD) being a common cause of PH.

PH is divided into five groups. Group 1, or PAH, has an incidence of 6 cases/million adults, a prevalence of 48 to 55 cases/million adults, and predominantly affects women.^3,4^ Group 2 is caused by left heart disease and affects 50% of patients with heart failure with preserved ejection fraction and 50% to 70% of patients with mitral and aortic valvulopathies. Group 3 is associated with lung disease, affecting between 7% and 30% of patients with advanced COPD.^3^ In idiopathic pulmonary fibrosis, 8% to 15% of patients have been reported to have PH on initial work-up, with the prevalence increasing to 30% to 50% in advanced and > 60% in end-stage interstitial lung disease. Group 4 (chronic thromboembolic pulmonary hypertension) is caused by chronic thromboembolism, with an incidence of 2 to 6 cases/million adults and a prevalence of 26 to 38 cases/million adults. Group 5 has multifactorial etiologies (Table 1).^3^

**Table 1:** Pulmonary hypertension groups. PAH: pulmonary arterial hypertension; PH: pulmonary hypertension; CCB: calcium channel blocker; LHD: left heart disease; PA: pulmonary artery


GROUP	SUBTYPES	GENERAL PREVALENCE	TREATMENT STRATEGIES

1 PAH	Idiopathic Heritable Associated with drugs/toxins Associated with connective tissues disease Venous/capillary involvement Persistent PH of newborn	Rare	PAH medications CCB (select patients) Lung transplantation

2 PH associated with LHD	Heart failure Valvular disease Congenital/acquired cardiovascular conditions	Very common	Treatment of LHD Consider PAH medications

3 PH associated with lung disease and/or hypoxia	Obstructive Restrictive Mixed obstructive/restrictive Hypoventilation syndromes Hypoxia without lung disease Developmental lung disorders	Common	Treat underlying lung disease Consider PAH medications

4 PH associated with pulmonary artery obstructions	Chronic thromboembolic Other PA obstructions	Rare	Pulmonary endarterectomy Balloon pulmonary angioplasty Consideration medications

5 PH with unclear/multifactorial etiology	Hematological Systemic Metabolic Chronic renal failure with or without dialysis Pulmonary tumor thrombotic microangiopathy Fibrosing mediastinitis	Rare	Treatment of underlying disease Consider PAH medications


Females are known to have a higher incidence of PAH, which is reflected globally across registries in the US, Europe, and Asia (Table 2).^4,5,6,7,8,9,10,11,12,13,14,15,16,17^ Female-to-male prevalence varies by ethnicity, with one paper citing 3.2:1 female-to-male predominance in Whites, 4.7:1 in Hispanics, and 5.5:1 in Blacks.^3^ Data from various registries showed that most patients enrolled with PAH were female, such as the French and Scottish registries (> 65%), US REVEAL registry (> 80%), German Registry COMPERA, Spanish and Latvian registries (> 73%), and Japanese registry (76%).^7,9,10,11,13,14^ However, despite female predominance, women had better outcomes compared with male patients, a finding consistently reflected across all registries.^6,7,8,9,10,11^ This observation has led to a phenomenon called the “estrogen paradox.”

**Table 2:** Registries of pulmonary hypertension. PAH: pulmonary arterial hypertension; CTEPH: chronic thromboembolic pulmonary hypertension; iPAH: idiopathic pulmonary artery hypertension; PoPH: portopulmoanry hypertension; CHD: congenital heart disease; CTD: connective tissue disorders; hPAH: heritable pulmonary arterial hypertension


REGISTRY	# OF CENTERS # OF PATIENTS	STUDY COHORT	FEMALE TO MALE PREDOMINANCE	OUTCOMES BY GENDER (95% CI)

REVEAL US (2006-2009)^9^	55 PAH: 3515	PAH, > 3 months	4.:1 F:M ratio	Not reported

Spanish REHAP (Retrospective 1998-2006, prospective 2007-2008)^10^	31 PAH: 866 CTEPH: 162	PAH, CTEPH, > 14 years	PAH: 51-90% female CTEPH: 60% female	Males: HR 1.38* (1.03-1.83)

Latvian (2007-2016)^11^	1 PAH: 130 CTEPH: 44	PAH, CTEPH, > 18 years	PAH: 73% female CTEPH: 61% female	Not reported

COMPERA (2007-2011)^13^	28 iPAH: 587	iPAH, > 18 years	18-65 years: 2.3:1 F:M ratio > 65 years: 1.2:1 F:M ratio	Males: HR 1.952* (1.264-3.016)

Japanese (2008-2013)^14^	8 PAH: 189	PAH, > 18 years	PAH: 76.2% female	Not reported

UK (2001-2009)^15^	7 PoPH: 110	PoPH, > 18 years	PoPH: 50.9% female	No significant difference in survival between genders

Scottish (1986-2001)^16^	All Scottish hospitals PAH: 374	PAH, > 16 years	PAH: 1.5-3:1 F:M ratio	Median survival: iPAH: males: 3.8 y; females: 5.6 y CHD-PAH: males: 5.9 y; females 4.9 y CTD-PAH: males: 4.5 y; females 2.6 y

French (2002-2003)^17^	17 PAH: 354	iPAH, hPAH, anorexigen PAH, > 18 years	Overall: Female: 61% 1.5:1 F:M ratio	Male: HR 1* Female: HR 0.375 (0.212-0.662, female)


*risk factor for mortality, all patients

## The Estrogen Paradox

The estrogen paradox has two major tenets. First, although animal models have shown mostly protective evidence of estrogen on lung vasculature, human females have a higher prevalence of PAH.^18,19^ Second, despite females being more susceptible to PAH, they have a better response to treatment and survival compared with men.^18,19^

Multiple animal studies have demonstrated the protective effects of estrogen. The three major animal models include the chronic hypoxia model, monocrotaline-induced PH model, and Sugen/hypoxia model.^18,19,20^ The chronic hypoxia model involves exposing rats to chronic hypoxia, leading to pulmonary vasculopathy. There are two major limitations of this model—once the animals are re-exposed to room air, their vasculopathy reverses; also, the pulmonary vasculature does not form vaso-occlusive or plexiform lesions.^19^ The monocrotaline-induced PH model involves injecting monocrotaline into rats, leading to a systemic inflammatory response that results in right ventricular (RV) hypertrophy and remodeling, which ultimately leads to frank RV failure and death.^19^ The Sugen/hypoxia model involves injecting rats with Su5416, a vascular endothelial growth factor receptor 2 antagonist that leads to proliferation of mitogenic substances involved in the development of PH; afterward, the rats are exposed to chronic hypoxia, leading to the development of permanent angio-obliterative pulmonary lesions and severe PH.^19,20^

Animal models have shown less severe PH in females, with females developing less hypoxic pulmonary vasoconstriction. Another study demonstrated that ovariectomized rats had more severe PH, but supplementing with estrogen improved the severity.^19,21^ Despite these animal studies, however, estrogen has played a conflicting role in the development of PH in humans, with some estrogen metabolites being implicated in the development of PAH while others have been shown to attenuate it.^21^ Some theories as to why animal models conflict with human results point to the radical differences between animal and human menstrual cycles leading to altered estrogen metabolism.^19^ It is also hypothesized that estrogen may have context-specific effects, depending on local concentrations in the pulmonary vasculature.^19,22^

## General Treatments

Treatments for PAH include prostacyclin analogues, such as epoprostenol (intravenous, or IV), Treprostinil (IV, subcutaneous, oral, inhaled formulations), selexipag (oral, IV), and iloprost (inhaled); endothelin receptor antagonists (oral) such as bosentan, ambrisentan, and macitentan; phosphodiesterase type 5 (PDE5) oral inhibitors such as sildenafil and tadalafil; and the guanylate cyclase stimulator, riociguat (oral).^23^ Combination therapy is frequently prescribed.^23^ Women with PAH have shown improved survival and better response to treatment than men, especially with endothelin receptor antagonists.^23^ It is hypothesized that women fare better due to improved hemodynamic parameters, including better cardiac index, lower pulmonary vascular resistance, superior RV function, and lower right atrial and mean pulmonary artery pressures.^19,21^ Currently, no PAH treatment specific for females exists despite the predominance of PAH in women.

Current evidence does not support a first-line monotherapy agent^24^; rather, dual combination therapy is recommended, and frequent follow-ups for symptom assessment is required. Acute vasoreactivity testing is recommended to evaluate the response to calcium channel blockers (CCBs) in idiopathic, heritable, and drug- and toxin-associated PAH. Responders should be started on progressively titrated doses of CCBs and monitored in follow-ups. High-risk patients who do not respond to CCBs should be considered for IV prostacyclin analogs. In general, patients who do not respond to maximum triple therapy should be referred for lung transplant evaluation.^24^

## Future Medications

Current therapies focus on targeting the prostacyclin, endothelin-1, or nitrous oxide pathways. Several novel medications are being studied in randomized trials that target novel pathways. One study using sotatercept, a novel fusion protein that binds to activins and growth differentiation factors, has shown a decrease in pulmonary vascular resistance (PVR) at 24 weeks compared with placebo, with females comprising 87% of study participants.^25^ The STELLAR study, a multicenter randomized controlled trial, showed an improvement in the 6-minute walk test in patients on sotatercept compared with patients on placebo; females made up 79% of study participants.^26^ Another medication, seralutinib, a novel inhibitor of multiple aberrant kinase signaling pathways involved in pulmonary artery remodeling, has completed a phase 2 trial.^27^ A novel dry-powder formulation of imatinib (PDGF inhibitor) is also being studied in a novel phase 2b/3 design as an addon therapy in PAH patients.^28^ As research in PH continues, new medications targeting different pathways will hopefully continue to improve patient outcomes.

## Special Considerations for Women

### Sexual Dysfunction

Sexual health is an important aspect of many patients’ lives, yet few studies have explored the effects of PH on intimacy in women. One study by Oliveria et al. showed that 71.8% of women with PH reported sexual dysfunction,^29^ while another study showed that 72% of partners of PAH patients reported a decrease in sexual engagement.^30^ Another study with 25 patients showed that patients’ concerns regarding intimacy included negative body image secondary to treatment modalities (catheters, pumps), changes in patients’ sense of femininity, a partner’s concern that they would harm the patient, low energy, and fear of pregnancy.^31^ The study also reported that intercourse was only spoken about from the context of avoiding pregnancy, and patients were ashamed to bring up the subject to their physician, especially if the physician was male. After patients had time to adjust to their new diagnosis, however, they reported improvement in their sexual life.^31^ Sexual health is a topic that needs to be approached in a sensitive manner as it can make a significant impact on a patient’s relationship with their partner.

### Contraception and Pregnancy

All guidelines recommend that patients with PAH avoid pregnancy and strongly consider termination should they become pregnant,^32^ because pregnancy brings about unique physiologic changes that can cause serious consequences in these patients. These physiologic changes include decreases in systemic vascular resistance, an increase in blood volume (40 - 100% from baseline), red cell mass (25% increase) and left ventricular mass, and heart dilation up to 30%.^32,33^ In PAH patients, compensatory mechanisms to accommodate vasodilation of the pulmonary vasculature are decreased or absent, leading to increased pulmonary vascular resistance.^32,34^ These changes lead to worsening RV strain and ultimately failure. Pregnancy also increases hypercoagulability and risk of thromboembolic events due to increases in coagulation factors and fibrinogen, decreased protein S, and more protein C resistance.^35^ Delivery and postpartum physiologic changes also bring about additional challenges, which are discussed further below.

Patients are strongly advised to use contraception, although natural family planning and barrier methods should not be used as the sole method of contraception.^32,36^ Estrogen-containing contraceptives and injected progestins increase the risk for thromboembolism and should be avoided. Progesterone-only pills could be used, and permanent contraception should be considered, although some patients understandably may not comply.^32,34,36^ Intrauterine devices can be considered but can cause vasovagal reactions during insertion and lead to RV strain. Two methods of contraception are recommended in general but are considered necessary when an endothelin receptor antagonist is used. Finally, in vitro fertilization and egg harvesting are not advised in PAH patients as they are associated with significant side effects, such as hyperstimulation syndrome and risk of venous thromboembolism, even in healthy patients.^32^ Patients with heritable PAH should undergo genetic counseling.^32,34,36^

### Treatment of Pregnant Women

It is highly recommended that pregnant patients be referred to a specialized treatment center for PAH,^36^ because a multidisciplinary approach is essential to help coordinate prenatal care, mode of delivery, anesthesia during delivery, and postpartum care. The multidisciplinary team (MDT) approach while managing these patients in a PH center has shown to improve maternal and fetal outcomes. One study conducted in China analyzed patients in pre- and post-MDT groups after the development of an MDT PAH center. It found that the pre-MDT group had higher rates of heart failure (30.6% vs 12.9% for pre- and post-MDT, respectively), higher rates of urgent Cesarean sections (C-sections) (41.6% vs 14.8%), less PAH-specific therapy (24.2% vs 59.5%) of which a majority was monotherapy rather than combination, and higher mortality rates (10.2% vs 0) than the post-MDT group.^37^ An MDT consisting of a high-risk obstetrician/gynecologist, cardiologist, critical care specialist, cardiovascular anesthesia specialist, and pulmonologist can help guide different aspects of a pregnant PAH patient’s care to help improve outcomes.^34,36,37^

The goal of treatment during pregnancy is to optimize RV function.^38^ Patients with severely depressed RV function should be started on IV epoprostenol during pregnancy, delivery, and postpartum; those with more preserved RV function may be carefully monitored and continued on oral therapies and/or considered for inhaled prostacyclins. Oral phosphodiesterase 5 inhibitors such as sildenafil should be continued in patients with normal RV function, and combination with parenteral prostaglandins has been reported to be successful.^36^ Patients with a positive vasodilator response and normal RV function can be carefully continued on CCBs, with close monitoring for deterioration. Certain oral agents such as endothelin receptor antagonists and soluble guanylate cyclase stimulator (riociguat) must be avoided due to their teratogenic effects.^24,36,39^ During pregnancy, patients should be monitored every 4 weeks with echocardiograms at each visit until the third trimester, at which point they should be followed with weekly clinical evaluation, including echocardiograms.^39^

It is recommended to stop endothelin receptor antagonists and riociguat, and strongly encouraged to stop selexipag due to their teratogenic effects.^24,36,29^ Though there is limited data, phosphodiesterase type 5 inhibitors, CCBs, and inhaled/IV/subcutaneous prostacyclin analogues are considered safe during pregnancy.

### Mode of Delivery

Delivery and the postpartum period are a particularly high-risk time for patients with PAH. During labor and delivery, dramatic hemodynamic and hormonal changes occur that can severely strain the RV. Immediately after delivery, 300 mL to 500 mL of blood is autotransfused back into maternal circulation due to uterine contractions. Additionally, venous flow improves as delivery relieves IVC obstruction caused by the gravid uterus. This leads to increased cardiac output that may take up to 48 hours to normalize, leading to RV strain and potentially RV failure.^39^ Patients should be advised to have a planned C-section instead of vaginal delivery as Valsalva maneuvers, subsequent vasovagal responses, and labor induction agents for vaginal delivery can worsen pulmonary vascular resistance and lead to cardiovascular collapse.^39^

During delivery, IV prostaglandin should be administered to most patients.^34,36^ Close monitoring of hemodynamics should be performed, with current guidelines recommending the use of a central venous catheter and arterial line; however, routine use of a Swan-Ganz catheter is not recommended.^34,36^ As medicine continues to advance, noninvasive methods may be used in the future. One case report of a patient with PH due to severe mitral valve stenosis was monitored via a Vigileo monitor during a planned C-section. The authors reported being able to optimize the patient’s fluid status accurately with a favorable outcome.^40^ Current guidelines recommend invasive monitoring for fluid and hemodynamic status.

### Postpartum Care

Postpartum PAH patients should be monitored in the intensive care unit (ICU) setting for several days since the immediate postpartum period carries a high rate of decompensation due to fluid shifts, hypercoagulable state, and catecholamine surge, resulting in death mainly from right heart failure, pulmonary embolism, and sudden death.^39^ Patients who decompensate should be treated with IV prostacyclin and monitored in the ICU setting. Patients should be anticoagulated with low-molecular-weight heparin to prevent thromboembolism and should continue to receive IV epoprostenol postpartum.^39^ Patients with improved hemodynamics and stable RV function postpartum can be transitioned back to their prepregnancy oral PAH medications and tapered off IV prostacyclin infusion. These changes in PAH medications require close monitoring to prevent any sudden hemodynamic compromise.

### Outcomes

Prior to the advent of prostacyclin as a medication for PAH, pregnant patients with PAH had higher rates of mortality (30 - 56%) and fetal death (11 - 28%).^32,34^ With the development of PAH-specific treatments, however, there have been improvements in maternal and fetal mortality rates,^32,34,41,42^ with more reports of successful pregnancy outcomes. Several small studies have reported maternal mortality rates of 16% to 17%.^38,41,42,43^ One study of seven pregnant women reported that patients treated for PAH prior to pregnancy had less RV dysfunction compared with newly diagnosed patients who had never received PAH treatment.^38^ Another study of 2,200 patients focused on pregnant women with congenital heart disease, with and without PH.^41^ Approximately 42% of patients had an abortion or miscarriage. Of those who successfully completed pregnancy, maternal mortality was 1 (0.1%), 0, and 19 (5.7%) in women with CHD with no, mild, or moderate-to-severe PH, respectively, with overall mortality 2.6% in pregnant women with CHD-associated PH. Overall, there were significantly lower rates of maternal and infant complications in patients with mild PH compared with moderate-to-severe PH. The most frequent infant complication was low birth weight (17.2%), which was more common in the moderate-to-severe PH group.^41^ A separate smaller study also demonstrated that pregnant PAH patients with a low PAH risk profile followed by MDT had improved outcomes,^44^ with outcomes summarized in Table 3.

**Table 3:** Outcomes of pregnant patients with PAH but with a low risk profile and followed by multidisciplinary team. PAH: pulmonary arterial hypertension; a/w: associated with; C: combination therapy; CHD: congenital heart disease; CS: C-section; CTD: connective tissue disease; GA: general anesthesia; G5: group 5; IS: intraspinal; LHD: left heart disease; MDT: multidisciplinary team; M: monotherapy; NA: no anesthesia; N/A: not applicable; iPAH: idiopathic PAH; oPAH: other PAH; oPH: other PH; PH: pulmonary hypertension; RA: regional anesthesia; SLE: systemic lupus erythematous; T: targeted therapy; VD: vaginal delivery


STUDY + NUMBER OF PREGNANT PH PATIENTS	PH ETIOLOGY	PH TREATMENT DURING PREGNANCY	DELIVERY WEEK (RANGE) AND MODE OF DELIVERY (PERCENTAGE)	TYPE OF ANESTHESIA	MATERNAL AND FETAL MORTALITY

Zhang et al. (1993-2016 retrospectively, 2017-2019 prospectively)^39^ 2220 total pregnancies 729 had PH	Carried pregnancy: No PH: 1491 Mild PH: 398 CHD-PAH: 346 (86.9%) CHD-oPH: 52 (13.1%) Moderate-to-severe PH: 331 CHD-PAH: 273 (82.5%) CHD-oPH: 58 (17.5%) Terminated pregnancy: 535 Mild PH: 41 Moderate-to-severe PH: 494	No PH: n/a Mild PH: T: 29 (7.33%) M: 26 (6.5%) C: 3 (0.8%) Moderate-to-severe PH: T: 99 (29.9%) M: 68 (20.5%) C: 31 (9.4%)	No PH: 37.8 + 1.98 weeks VD: 246 (16.8%) CS: 1218 (83.2%) Mild PH: 37.4 + 2.1 weeks VD: 44 (11.1%) CS: 354 (88.9%) Moderate-to-severe PH: 35.6 + 3.1 weeks VD: 26 (7.9%) CS: 305 (92.1%)	No PH: GA: 36 (2.5%) RA: 1209 (82.6%) NA: 219 (15%) Mild PH: GA: 9 (2.3%) RA: 347 (87.2%) NA: 42 (10.6%) Moderate-to-severe PH: GA: 38 (11.5%) RA: 273 (82.5%) NA: 19 (5.7%)	Maternal mortality: No PH: 1 (0.1%) Mild PH: 0 (0) Moderate-to-severe PH: 19 (5.7%) Fetal mortality: 535 (42.3%) miscarriages or terminations in PH patients Mortality: No PH: 7 (0.5%) Mild PH: 1 (0.3%) Moderate-to-severe PH: 4 (1.2%)

Lv C et al. (2011-2020)^41^ 154 pregnant PH patients	Carried pregnancy: 139 iPAH: 6 (3.9%) CHD-PAH: 34 (82.9%) oPAH: 40 (88.9%) LHD-PH: 59 (95.2%) Terminated pregnancy: 15 iPAH: 0 CHD-PAH: 7 (17.1%) oPAH: 5 (11.1%) -LHD-PH (3 (4.8%)	NR	Delivery range: Total: 30.9 + 5.1 weeks iPAH: 28.7 + 4.5 weeks CHD-PAH: 30.6 + 4.6 weeks oPAH: 30.9 + 4.6 weeks LHD-PH: 31.5 + 5.7 weeks Mode: VD: 29 (18.8%) CS: 108 (70.1%)	Patients who underwent CS: GA 52 (33.8%) IS: 56 (36.4%)	Maternal mortality: Total: 5 (3.2%) iPAH: 3 (50%) CHD-PAH: 1 (2.4%) oPAH: 0 (0) LHD-PH: 1 (1.6%) Fetal mortality: Therapeutic abortion: 15 (9.7%) Missed abortion: 1 (0.6%) Intrauterine death: 7 (4.5%) Neonatal death (< 1 week): 3 (1.9%)

Chen et al. (2004-2020)^35^ 103 pregnant PH patients	Pre-MDT: 49 iPAH: 1 (2.0%) CTD-PAH: 2 (4.1%) CHD-PAH: 30 (61.2%) LHD-PH: 14 (28.6%) G5-PH: 2 (4.1%) Post-MDT: 54 iPAH: 3 (5.6%) CTD-PAH: 1 (1.9%) CHD-PAH: 33 (61.2%) LHD-PH: 12 (22.2%)	Pre-MDT: T: 8 (24.2%) M: 7 (87.5%) C: 1 (12.5%) Post-MDT: T: 22 (59.5%) M: 12 (54.5%) C: 10 (45.5%)	Pre-MDT: 37 (31, 42) weeks VD: 2 (4.2%) CS: 46 (95.8%) Post-MDT: 36 (27, 40) weeks VD: 1 (1.9%) CS: 533 (98.1%)	Pre-MDT: GA: 18 (39.1%) IS: 28 (60.9%) Post-MDT: GA: 38 (71.7%) IS: 15 (28.3%)	Maternal mortality: Pre-MDT: 5 (10.2%) Post-MDT: 0 (00%) Fetal mortality: Abortions/terminations: not reported Mortality: Pre-MDT: 7 (14.2%) Post-MDT: 1 (1.9%)

Corbach et al. (2004-2020)^42^ 5 patients, 7 pregnancies	iPAH: 3 (60%) PAH a/w SLE: 1 (20%) PAH a/w schistosomiasis: 1 (20%)	CCB: 4 pregnancies (57%) PDE-5: 7 pregnancies (100%)	37.1 (33.7, 38.0) weeks CS: 7 (100%)	IS: 7: 100%	Maternal mortality: 0 (0%) Fetal mortality: Abortions/terminations: 0 Mortality: 0 (0%)

Vaidya et al. (2013-2021)^36^ 6 patients, 10 pregnancies	Completed pregnancy: 6 iPAH: 3 (50%) CHD-PAH: 2 (33.3%) SLE-PAH: 1 (16.7%) Terminated pregnancy: 3 hPAH: 1 (33.3%) CHD-PAH: 1 (33.3%) oPAH: 1 (33.3%)	Prepregnancy: None: 2 (28.6%) Ambrisentan + sildenafil: 3 (42.8%) Treprostinil: 1 (14.3%) Tadalafil: 1 (14.3%) Pregnancy:Sildenafil/Tadalafil + treprostinil: 6 (85.7%) Tadalafil: 1 (14.3%) Epoprostenol at delivery: 2 (28.6%)	36.9 (31.3, 40.4) weeks VD: 3 (42.9%) CS: 4 (57.1%)	GA: 1 (14.3%) IS: 6 (85.7%)	Maternal mortality: 0 (0%) Fetal mortality: Abortions: 3 (30%) Mortality: 0 (0%)

Duarte et al. (1999-2009)^43^ 18 pregnant PH patients	Completed pregnancy: 12 iPAH: 4 (33.3%) CTD-PAH: 2 (16.7%) CHD-PAH: 6 (50%) Terminated pregnancy: 6 iPAH: 3 (50%) CHD-PAH: 2 (33.3%) Stimulant: 1 (16.7%)	Completed pregnancies: Prepregnancy: None: 7 (25%) ERA: 3 (25%) Prostaglandin: 1 (8.3%) CCB: 1 (8.3%) During pregnancy: None: 3 (25%) Sildenafil: 3 (25%) Prostanoid: 5 (41.7%) Combination: 1 (8.3%) Terminated pregnancies: None: 1 (16.7%) ERA + PD: 3 (50%) ERA: 2 (33.3%)	34 (28, 36) weeks CS: 12 (100%)	GA: 3 (25%) IS: 8 (66.7%) N/A: 1 (8.3%)	Maternal mortality: 1 (8.3%) Fetal mortality: Abortions: 6 (33.33%) Mortality: 0 (0%)


While the advent of PAH-specific therapies has improved maternal and fetal outcomes, it must still be emphasized that PH remains a devastating illness that becomes highly complicated during pregnancy, even with the help of MDTs.^45^ The current guidelines continue to emphasize the need to avoid pregnancy.

### Lactation

There is limited data regarding the transfer of PAH medications to breast milk and limited or unknown data on endothelin receptor antagonists, iloprost, epoprostenol, and treprostinol.^46^ Sildenafil is known to transfer to breast milk.^46,47,48^ One case report of a mother treated with sildenafil and bosentan reported low medicine concentrations in her breast milk 21 months after delivery, with optimal health of the nursing infant, who was not exclusively breastfed.^47^ Multiple other case reports have shown no overt effects in breastfed infants with mothers treated with sildenafil and bosentan.^49,50,51^ The decision to breastfeed should be made after a careful discussion between the patient and her physician.

## Conclusion

PH is a rare condition, with PAH having a strong female predominance. Special consideration must be taken with female patients as they have unique challenges with family planning, response to therapy, and survival. In addition, research is needed to help tailor PH treatment for female patients.

## Key Points

- Females have a higher incidence of pulmonary arterial hypertension compared with men but have better outcomes, leading to a phenomenon called the estrogen paradox.
- Special considerations should be given to women with PAH in areas such as sexual health, contraception, family planning, and disease management during pregnancy.
- Pregnancy, especially postpartum, remains a critical time for female patients with PAH, and patients should ideally be treated at PAH centers by a multidisciplinary team.
